# Modeling and optimizing concentration of exogenous application of *γ-*aminobutyric acid on NaCl-stressed pineapple mint (*Mentha suaveolens*) using response surface methodology: an investigation into secondary metabolites and physiological parameters

**DOI:** 10.1186/s12870-023-04312-w

**Published:** 2023-06-10

**Authors:** Hosein Ahmadi, Habib Farhadi, Mohammad Reza Morshedloo, Filippo Maggi

**Affiliations:** 1 Department of Horticulture Science, College of Agriculture and Natural Resources, University of Tehran, P.O.Box 31587 77871, Karaj, Iran; 2 Department of Horticultural Science, Faculty of Agriculture, University of Maragheh, P.O.Box 55136-553, Maragheh, Iran; 3 Center of International Scientific and Collaborations (CISSC), Ministry of Science, Research and Technology, Tehran, Iran; 4 School of Pharmacy, Chemistry Interdisciplinary Project (ChIP), University of Camerino, Camerino, 62032 Italy

**Keywords:** Pineapple mint, *Mentha suaveolens*, Piperitenone oxide, Response surface methodology

## Abstract

Salinity, a severe worldwide issue, compromises the economic production of medicinal plants including mints and causes drug-yield decline. γ-Aminobutyric acid (GABA) is a tolerance-inducing signaling bio-molecule in various plant physiological processes. Pineapple mint (*Mentha suaveolens* Ehrh.) is a valuable medicinal herb with an exhilarating scent of citrus fruit. Piperitenone oxide is the major bioactive constituent of its essential oil, having significant demand by pharmaceutical industries. Nonetheless, modeling and optimizing the effective concentration of GABA remain within twin foci of interest. Therefore, a two factor-five level (NaCl 0-150 mM and GABA 0-2.4 mM) central composite design was conducted to model and optimize drug yield and physiological responses of *M*. *suaveolens*. Based on the design of experiments (DoE) approach, different linear, quadratic, cubic, and quartic models were assigned to the response variables. Change trends of shoot and root dry weights followed a simple linear model, whereas sophisticated models (i.e., multiple polynomial regression) were fitted to the other traits. NaCl stress inevitably reduced root and shoot dry weight, piperitenone oxide content, relative water content, pigments content, and maximum quantum yield of PSII. However, content of malondialdehyde (MDA) and total flavonoid, and DPPH radical scavenging activity were increased under salinity. Under severe NaCl stress (150 mM), the essential oil content (0.53%) was increased three times in comparison with control (0.18%). Optimization analysis demonstrated that the highest amount of essential oil (0.6%) and piperitenone oxide (81%) as a drug yield-determining component would be achievable by application of 0.1–0.2 mM GABA under 100 mM NaCl. The highest dry weight of root and shoot was predicted to be achieved at 2.4 mM GABA. Overall, extremely severe NaCl stress (i.e., more than 100 mM) in which a sharp drop in yield components value was observed seemed to be out of *M. suaveolens* salinity tolerance range. Hence, it is rationale to compensate the decrease of drug yield by foliar application of a dilute GABA solution (i.e., 0.1–0.2 mM) under 100 mM NaCl stress or lower levels.

**Supplementary Information:**

The online version contains supplementary material available at 10.1186/s12870-023-04312-w.

## Background

The genus *Mentha* (Lamiaceae) comprises over 60 species, broadly distributed in temperate and semi-temperate zones of the world [1]. *Mentha suaveolens* Ehrh. cv. *variegata*, commonly known as Pineapple mint, is one of the several cultivars of this sopecies possessing bumpy and woolly leaves surrounded by a white margin [2]. Pineapple mint mainly grows in humid places and is often planted as an ornamental plant, fragrant groundcover, and sometimes found in hanging baskets [3].

Essential oils (EOs) from mint species are economically important natural products, presented among the top 10 traded Eos list. In a prediction, the business of these commodities is expected to reach a turnover of more than 27 billion US dollars by the end of 2022 (www.statista.com). Indeed, EOs contain a vast range of pharmaceutical attributes and are constantly used industrially throughout the world [4]. The EO of pineapple mint is a rich source of piperitenone oxide, an oxygenated monoterpene with an extensive range of beneficial effects such as anti-nociceptive, analgesic, antimicrobial, and insect-repellent [5]. Much literature has reported that EOs and drug yield components of different medicinal species are substantially up-regulated or down-regulated under a multitude of environmental stresses [6, 7]. The reverse aspect of this phenomenon is one of the primary obstacles to the high-throughput production of medicinal plants [8]. Some scholars have corroborated that secondary metabolites are often accumulated as compatible solutes to keep the standard water potential of plant cells under osmotic stresses [7, 9, 10]. The biosynthesis of secondary metabolites is undoubtedly regulated by different gene expression networks, enzymes, transcription factors, and various kinds of abiotic stresses, which ultimately trigger a complicated defense mechanism through a wide range of signaling molecules to survive in unfavorable conditions [7, 11, 12].

Nowadays, soil salinization is a critical abiotic stress occurring whether naturally or an artificial process has become an alarmingly critical problem, limiting the sustainable production of crops in 10% of the world lands as well as more than 30% of soil surfaces in Iran [13]. Salinity can impress plant growth through a drastic ionic imbalance, ion toxicity (excessive amounts of Na^+^ and Cl^−^), oxidative burst, pigments degradation, restricting flux of CO_2_, and inhibiting K^+^ uptake as an essential element for plant development [14, 15]. As a result of an ionic imbalance in plant cells and magnesium deficiency, the biosynthetic pathway of chlorophylls is also diverted [14]. These metabolic abnormalities annihilate the structure and efficiency of photosystem II. Thus, a severe diminishment in maximum quantum yield of PSII and total biomass production is expected [14]. Despite the slow progress of ongoing breeding programs, the alleviation of salt-induced damage through foliar application of amino acids has become a fast and alternative method to prevent osmotic injuries in horticultural crops through providing sufficient carbon and nitrogen skeleton during nutritional starvation as well as by activating signaling pathways, ion transport and the related metabolism [13]. Gamma-aminobutyric acid (GABA), a ubiquitous non-proteinogenic amino acid, links two primary metabolic pathways of carbon and nitrogen, and is believed to function as a tolerance-inducing signaling bio-molecule in various physiological processes [16]. GABA is also a safe health-promoting neurotransmitter showing relaxing and anti-hypertension impacts [17]. This versatile amino acid has attracted considerable attention of the food and pharmaceutical industries, and nowadays GABA-enriched food stocks have been globally commercialized [17]. In the plant kingdom, the GABA shunt pathway is involved in the biosynthesis of *γ-*aminobutyric acid from glutamate [18]. Aghdam et al. [19] showed that GABA permease, a mitochondrial GABA transporter, carries GABA from the cytosol into mitochondria to catalyze the reaction of GABA conversion to succinic semialdehyde through another enzyme known as GABA transaminase. Subsequently, succinic semialdehyde is converted to succinate, which can enter the TCA cycle and donate the electron to the mitochondrial electron transport chain to support sufficient ATP production and decline ROS accumulation [19]. It has been proposed that an improved stress tolerance in horticultural crops by GABA application can be attributed to 1) higher endogenous GABA accumulations; 2) higher endogenous energy (ATP) status; 3) higher activity of the antioxidant system accounting for lower ROS accumulation; 4) higher proline accumulation due to higher pyrroline-5-carboxylate synthetase and lower proline dehydrogenase activity; 5) higher accumulation of phenolic compounds due to higher phenylalanine ammonia-lyase/polyphenol oxidase enzyme activity ratio [14, 20].

Response surface methodology (RSM) comprises mathematical models and statistical techniques used to process the optimization of a response variable with the fewest experiments required [21, 22]. On the other hands, RSM includes optimization procedures for the settings of variables, such that the response reaches a desired value of treatments. This methodology requires fewer experimental runs than full factorial designs but delivers statistically satisfying results [21]. Central composite design (CCD) is one of the most popular designs of experiment approaches based on response surface methodology as many scholars have frequently applied it for modeling and optimizing purposes in their experiments, such as extraction of plants’ secondary metabolites [12, 22–25], plant nutrition studies [26], and elicitor applications [27].

In the present study, modeling and optimizing the suitable concentration of GABA to achieve the highest dry weight yield, essential oil and piperitenone oxide contents, and physiological parameters has been conducted using response surface methodology. In the meantime, the theoretically optimized concentration of GABA accomplished by this study will be applicable in a high-throughput production of medicinal plants and other agricultural practices under salinity stress.

## Material and methods

### Chemicals and reagents

γ-Aminobutyric acid (GABA) was purchased from Sigma–Aldrich company (USA). Thiobarbituric acid (TBA), AlCl_3_, HCl, NaHCO_3_, HPLC grade methanol and GC grade *n*-hexane were purchased from Merck company (Darmstadt, Germany).

### Experimental design using response surface methodology

**Fig. 1:**
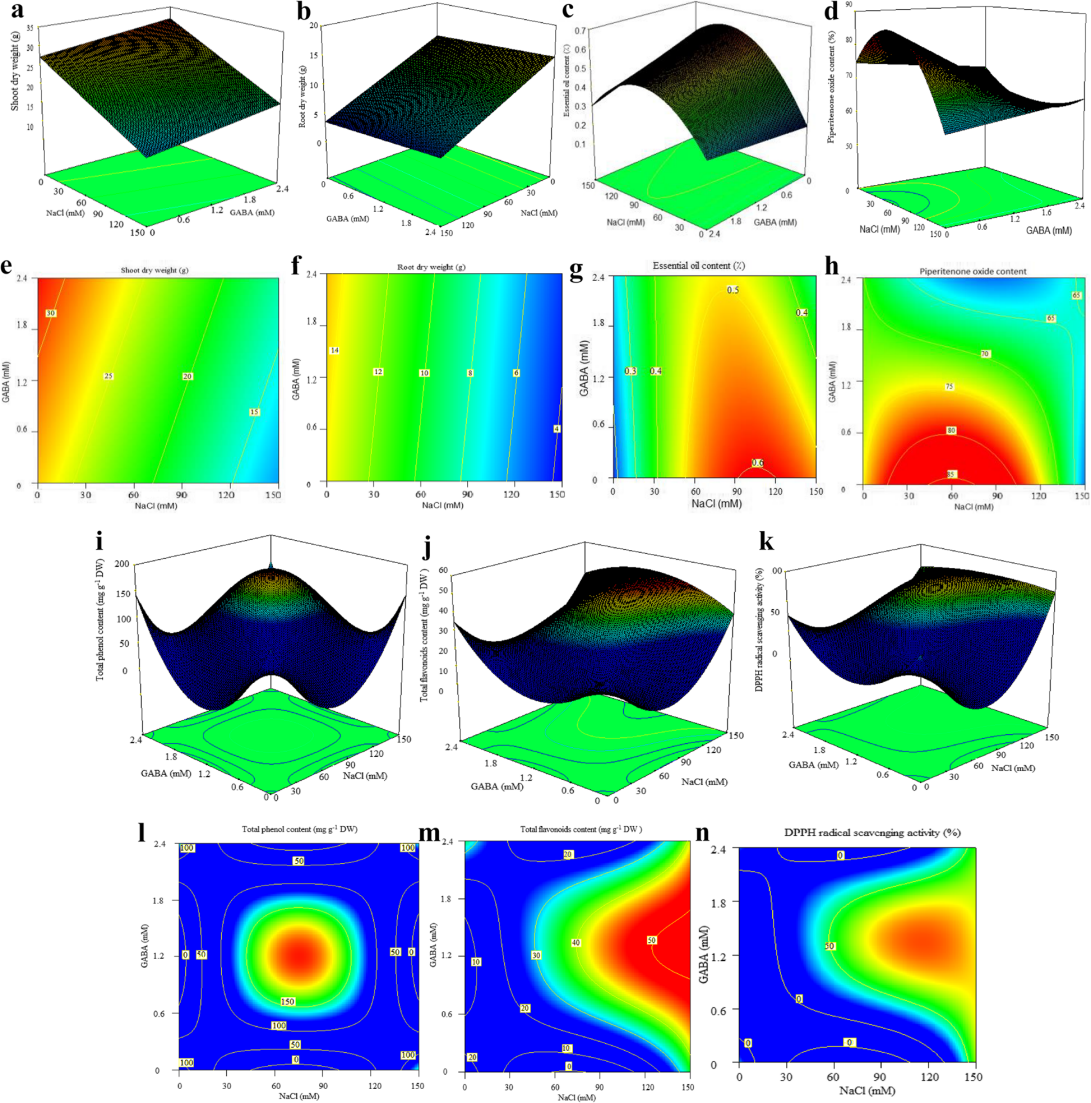
Response surface plots (3D and contour plots) of yield-related traits (dry materials yield, essential oil content and piperitenone oxide content) and antioxidant indices under NaCl stress and GABA application. The plots show the effect of GABA concentration and salinity stress on measured traits

**Fig. 2:**
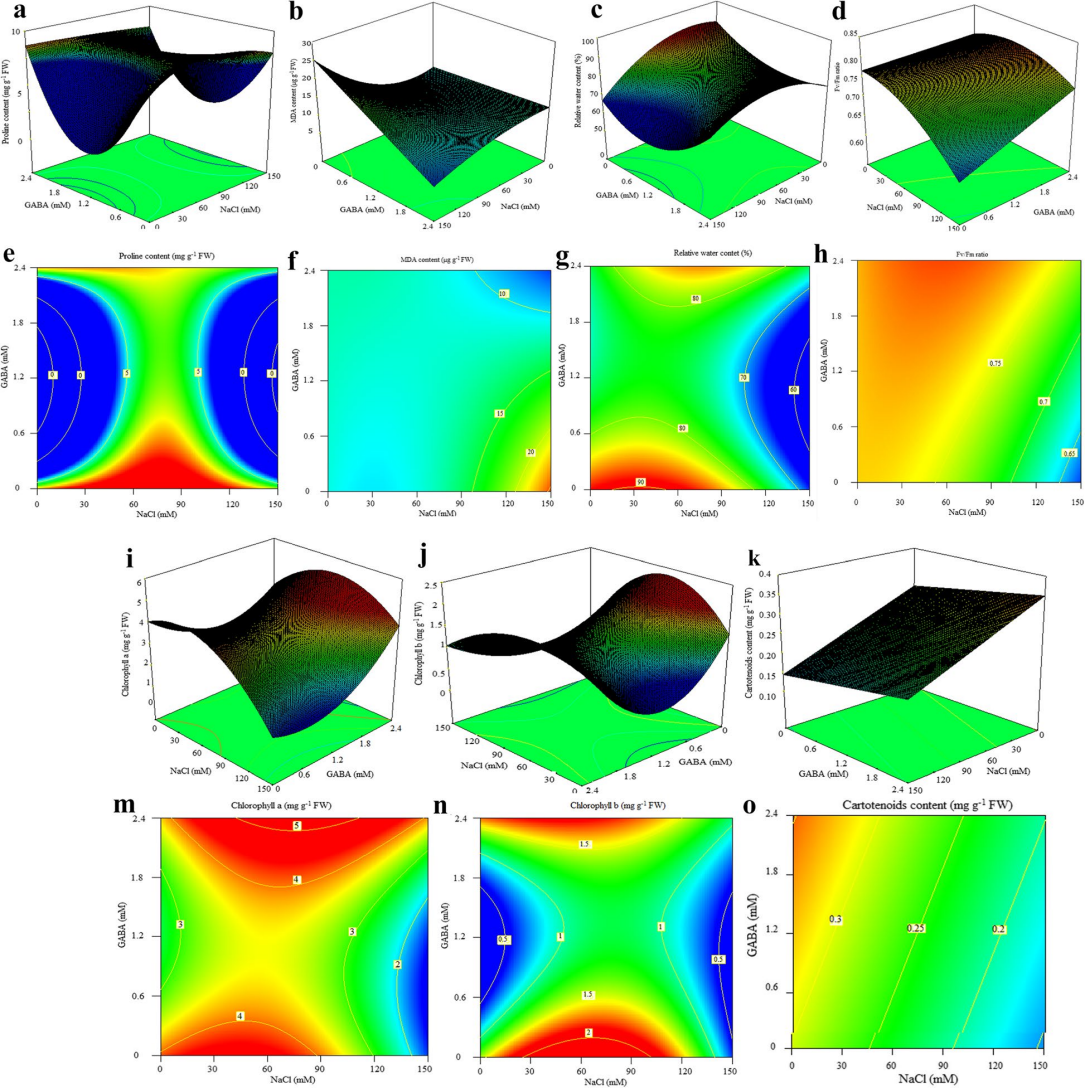
Response surface plots (3D and contour plots) of physiological parameters under NaCl stress condition and GABA application. The plots show the effect of GABA concentration and salinity stress on measured traits

All experimental variables were measured following a pre-determined arrangement of a 30-run central composite design (CCD) consisting in two independent variables (NaCl and GABA treatment), each with five levels explained in details in Table 1. The selected value for α was 0.3333. Table 1 displays the entire set of experimental runs, consisting in four factorial points (plus and minus one), four axial points (plus and minus alpha), and one center points (zero). Note that three replications were assigned to axial and factorial points, and six replications were allocated to central point (i.e., 75 mM NaCl and 1.2 mM GABA). The data obtained from dependent variables were subjected to response surface regression analysis using the least square methodology to determine the equations of actual factors fitting multiple linear or polynomial models. The adequacy of each model was examined by evaluating the lack of fitness, coefficient of regression (R^2^), precision adequacy, and F-value through variance analysis (ANOVA). Moreover, the other diagnostic tests were evaluated for further evidence of the goodness of fit in the provided models. When needed, the statistical significance level of some response variables (reduced models) was improved through a backward-elimination method by removing the insignificant terms (*p* > 0.05) using algorithmically multiple model selection criteria. Response surface and contour plots were also obtained using the fitted models presented in Figs. 1 and 2. Furthermore, Duncan’s multiple ranges mean comparison test (DMRT) was carried out based on variance analysis of a completely randomized design (CRD) consisting in 9 treatments. Using xlstat 2018, principal component (PCA) and cluster (CA) analyses were conducted to unveil dissimilarities and behavior of response variables under GABA and NaCl treatments. Using mean values, R studio software (2022) helped to construct an interactive heatmap plot displaying the relative correlations between measured traits.

**Table 1:** Experimental values measured for each trait (*n* = 30) based on central composition design (CCD)

Traits	NaCl (mM)	0 (-1)	150 (+ 1)	0 (-1)	150 (+ 1)	50 (-α)	100 (+α)	75 (0)	75 (0)	75 (0)
	GABAm(mM)	0 (-1)	0 (-1)	2.4 (+ 1)	2.4 (+ 1)	1.2 (0)	1.2 (0)	0.8 (-α)	1.6 (+α)	1.2 (0)
Total flavonoids content (mg GAE g^− 1^ DW)		26.51 ± 0.91 g	36.24 ± 1.29 de	35.43 ± 0.79 de	41.29 ± 1.49 b	31.1 ± 0.36 f	46.2 ± 0.57 a	34.12 ± 1.36 e	38.09 ± 1.80 cd	39.94 ± 0.57 bc
Total phenols content (mg QE g^− 1^ DW)		144.51 ± 3.91 c	143.75 ± 5.54 c	143.5 ± 3.64 c	145.03 ± 3.89 c	167.06 ± 1.03 b	167.4 ± 1.58 b	168.29 ± 2.25 b	166.4 ± 1.26 b	191.27 ± 1.23 a
DPPH-scavenging (%)		31.06 ± 2.8 c	66.83 ± 10.16 b	28.88 ± 5.96 c	59.48 ± 3.06 b	39.28 ± 4.1 c	79.18 ± 1.47 a	41.09 ± 9.53 c	62.94 ± 3.25 b	64.99 ± 2.84 b
Proline content (mg g^− 1^ FW)		6.66 ± 0.19 b	6.13 ± 0.22 c	7.41 ± 0.16 a	5.92 ± 0.09 c	4.27 ± 0.07 e	5.06 ± 0.05 d	6.59 ± 0.19 b	6.07 ± 0.21 c	6.16 ± 0.07 c
Relative water content (%)		88.74 ± 0.03 a*	67.27 ± 0.73 e	76.21 ± 1.52 bc	76.12 ± 0.93 bc	77.53 ± 0.41 b	71.39 ± 0.55 d	76.44 ± 0.44 bc	77.18 ± 0.01 b	75.2 ± 0.43 c
MDA content (mg g^− 1^ FW)		11.27 ± 0.77 c	25.37 ± 1.69 a	12.51 ± 0.59 bc	6.26 ± 1.40 d	11.37 ± 0.57 c	13.44 ± 0.47 b	12.01 ± 0.85 bc	11.61 ± 1.03 bc	11.33 ± 0.35 c
Chlorophyll a content (mg g^− 1^ FW)		4.08 ± 0.22 a	1.47 ± 0.11 d	3.81 ± 0.39 abc	3.91 ± 0.26 ab	3.52 ± 0.14 bc	3.41 ± 0.13 c	3.71 ± 0.33 abc	3.82 ± 0.06 abc	3.39 ± 0.13 c
Chlorophyll b content (mg g^− 1^ FW)		1.4 ± 0.04 b	0.61 ± 0.06 e	1.76 ± 0.13 a	1.07 ± 0.01 cd	1.02 ± 0.06 d	1.07 ± 0.06 cd	1.32 ± 0.05 b	1.18 ± 0.04 c	1.12 ± 0.04 cd
Carotenoids content (mg g^− 1^ FW)		0.28 ± 0.01 b	0.14 ± 0.04 e	0.35 ± 0.02 a	0.19 ± 0.03 d	0.25 ± 0.01 bc	0.23 ± 0.01 c	0.27 ± 0.01 b	0.26 ± 0.04 bc	0.26 ± 0.01 bc
Fv/Fm ratio		0.77 ± 0.02 a	0.62 ± 0.01 b	0.78 ± 0.01 a	0.74 ± 0.03 a	0.77 ± 0.01 a	0.75 ± 0.01 a	0.76 ± 0.01 a	0.76 ± 0.03 a	0.76 ± 0.01 a
Shoot dry weight (g)		27.7 ± 1.47 a	10 ± 1.90 d	30.33 ± 1.56 a	16.87 ± 1.35 c	22.23 ± 0.28 b	19.67 ± 0.69 bc	23.13 ± 3.07 b	22.83 ± 2.32 b	23.13 ± 1.03 b
Root dry weight (g)		14.93 ± 2.35 a	4.02 ± 0.4 c	15.45 ± 2.60 a	5.05 ± 0.33 c	9.15 ± 1.29 b	8.42 ± 1.14 b	8.38 ± 1.28 b	9.25 ± 0.54 b	8.42 ± 0.80 b
Essential oils content (%)		0.18 ± 0.03 e	0.53 ± 0.06 a	0.25 ± 0.05 cd	0.30 ± 0.05 c	0.42 ± 0.00 b	0.55 ± 0.03 a	0.52 ± 0.04 a	0.53 ± 0.06 a	0.56 ± 0.02 a
Piperitenone oxide content (%)		74.82 ± 3.27 ab	61.17 ± 3.7 e	70.24 ± 3.19 bcd	65.92 ± 5.24 de	72.87 ± 1.06 abc	70.21 ± 2.09 bcd	76.49 ± 3.79 a	68.54 ± 2.09 cd	74.7 ± 1.49 ab

* In each row means with the same letter do not have a significant difference (*p <* 0.05) with each other according to duncam mean comparison test

### Establishment of experimental runs and treatments

The present study was conducted in a glass greenhouse in the Faculty of Agriculture, University of Maragheh, Iran. Vegetative propagation was carried out as the first step to warrant genetically uniformity of plant materials. In this regard, rhizomes were cultured in 24-cell transplant trays containing 50:50 (v/v) coco peat: perlite medium. Transplants reaching ten pair-leaves stages (10–15 cm) were transferred into 10 L pots containing sieved field soil, sand, and leaf mold (2:1:1). The results of soil analysis are provided in a supplementary table (Table S1). Plants were kept at 18–30 °C; photoperiod of 16 h, relative humidity of 50–80%, and a nutritional program was exerted to warrant a sufficient nutritional support. One month after planting, mint clones (30 pots) were exposed to 5 different levels of salinity stress, including control (water), 30, 60, 90, 120, and 150 mM NaCl. The irrigation with NaCl solution (200 mL for each pot) was performed every 3 days. The total amount of NaCl consumed solutions per pot during the experiment was 6 L. To avoid osmotic shock, the NaCl treatments were initiated with a 20 mM NaCl solution, and then the concentrations were progressively enhanced (every two days) to reach the maximum values determined for each treatment.

Moreover, foliar application of GABA started five days before salinity treatments, and totally five different concentrations (control, 0.8, 1.2, 1.6, and 2.4 mM) were applied. Control treatments were subjected to foliar application of distilled water. Each pot per week was irrigated with 0.5 L of distilled water to avoid over-accumulating salt minerals. To prevent nutritional deficiency, plants were fed a one-fourth strength Hogland solution once every two weeks and during the growing season [28]. Finally, enough plant materials were kept at ˗ 80^°^C for subsequent biochemical analyses, and the remaining plant materials were oven-dried at 40^°^C and stored until EO and total polyphenols were extracted.

#### Assessment of relative water content and dry weights

At the full flowering stage, all plant parts were fully harvested. Then, roots and shoots were separated, dried, and weighted. To measure changes in leaf relative water content (RWC) in response to salinity stress treatments, 10 fully extended leaves were obtained from the plants, weighed with a precise scale for their fresh weight, and immersed in double distilled water for 24 h at 4^°^C until leaves became fully turgid. Next, the leaves were quickly weighed and placed in the oven at 70^°^C for 48 h. At the final step, the leaves were weighed again for their dried leaf weight [29]. The relative water content was determined from the following Eq. (1):

1 $$\%\mathrm{RWC}\;=\;\lbrack({\mathrm W}_{\mathrm f}\; - {\mathrm W}_{\mathrm d})/({\mathrm W}_{\mathrm t}\; - {\mathrm W}_{\mathrm d})\rbrack\times100$$

Where W_f_ denotes the fresh weight of leaves, W_t_ denotes the leaf’s turgid weight, and W_d_ denotes the dry weight of leaves.

### Proline content

The proline content of fresh leaves was estimated using a ninhydrin reagent [30]. Following the procedure, total proline content was extracted from the toluene phase and prepared for spectrophotometric measurements in a 96-well quartz plate. Finally, measurements were done by a plate reader at 520 nm. Total proline content was expressed as µmol proline g^˗1^ fresh weight.

### Malondialdehyde (MDA) content

Malondialdehyde (MDA) content as an indicator of membrane integrity was determined in the fresh leaves based on the method proposed by Heath and Packer [31]. Accordingly, thiobarbituric acid (TBA) reagent was used to capture the absorbance of the MDA-TBA adduct at 532 and 600 nm. Then, MDA content was determined using its extinction coefficient of 155 mM cm^− 1^. The content of MDA was expressed as nmol g^− 1^ fresh weight (FW).

### Chlorophylls and carotenoids content

Total chlorophyll *a*, chlorophyll *b*, and carotenoid contents were estimated according to the method described by Arnon [32]. For this purpose, 0.2 g of fresh leaf samples were extracted in 10 mL 80% acetone solution, and then samples were centrifuged for 15 min at 10,000 rpm. The absorption spectra of pigment extracts were recorded at 663, 645, and 470 nm, and total contents were calculated using the following Eqs. (2, 3, 4):

2 $$\mathrm{Chlorophyll}\;a\;\mathrm{content}\;(\mathrm{mg}\;\mathrm g^{-1}\;\mathrm{FW})\;=\;\lbrack12.7\;(\mathrm A663)\:-\:2.69\;(\mathrm A645)\rbrack\;\times\mathrm V/1000\;\mathrm W$$

3 $$\mathrm{Chlorophyll}\;b\;\mathrm{content}\;(\mathrm{mg}\;\mathrm g^{-1}\;\mathrm{FW})\;=\;\lbrack22.9\;(\mathrm A645)\:-\:4.68\;(\mathrm A663)\rbrack\;\times\mathrm V/1000\;\mathrm W$$

4 $$\mathrm{Carotenoids}\;\mathrm{content}\;(\mathrm{mg}\;\mathrm g^{-1}\;\mathrm{FW})\;=\;\lbrack(\mathrm A480)\;+\;(0.114\;\times\;\mathrm A663)\;-\;(0.638\;\times\;\mathrm A645)\rbrack\;\times\mathrm V/1000\;\mathrm W$$

V represents the consumed acetone volume in these equations, and W represents fresh leaves’ weight.

### Chlorophyll fluorescence

Chlorophyll fluorescence parameters (F_0_, F_m_, F_v_, F_v_/F_0_, and F_v_/F_m_) were recorded using a portable chlorophyll fluorescence meter (Pam 2500-Walz, Germany) between 9 and 11 a.m. Minimal fluorescence (F_0_) was automatically measured after 30 min dark-adaption of leaves, and maximal fluorescence (F_m_) was measured in the same light-adapted leaves.

### Total phenols and flavonoids content

Hydro-alcoholic extraction and the subsequent biochemical assessments were conducted using the outlines explained by Ahmadi et al. [33] with slight modifications. This assay blended 0.2 g of powdered dried leaves with 10 mL of methanol 80%. The protocol was completed by shaking samples at 200 rpm for 12 h. After centrifugation, the supernatants were separated, and then the residuals were re-extracted for the second time for 24 h.

Total polyphenol contents were measured using 10-times diluted Folin-Ciocalteu’s reagent and a 3% solution of NaHCO_3_. The absorbance values were recorded at 765 nm. The total content of polyphenols was expressed in mg gallic acid equivalent (GAE) per g^− 1^ dried weight [33].

The total content of flavonoids was estimated using the AlCl_3_ reagent according to the protocol that Ahmadi et al. [33] described. The investigation continued with reading the absorbance of samples at 415 nm. Total flavonoids of samples were finally quantified using the plotted calibration curve of quercetin. The total amount of flavonoids was expressed in mg quercetin equivalent (QE) per g^− 1^ dried weight.

### DPPH radical scavenging activity

Determination of 2,2-diphenyl-1-picrylhydrazyl (DPPH) free radical scavenging activity of hydro-alcoholic extracts was performed using a method reported in our previous studies [33, 34]. In this antioxidant assay, extracts were mixed with 0.3 mM methanolic solution of DPPH for 30 min in a dark room. The absorbance of examined samples was read at 517 nm. The experimental values were expressed in terms of percentage inhibition of DPPH radicals and calculated using Eq. (5).

5 $$\%{\mathrm I}\ =\ 100\ \times\ ({\mathrm{blank}}\ {\mathrm{absorbance}}\ -\ {\mathrm{sample}}\ {\mathrm{absorbance}})/{\mathrm{Absorbance}}\ {\mathrm{blank}}$$

### Essential oil content

Flowering aerial parts of each plant obtained from 30 experimental runs were separated and then subjected to an extraction process following the procedure by Ahmadi et al. [33]. Fifty g of the dried mixture of flowers and leaves were hydro-distilled using a Clevenger apparatus for about 3 h. Then, the redundant water was removed from the EO samples using anhydrous sodium sulfate. The content of EOs (%v/w) was determined by calculating the ratio of EO volume and dry weight of plant samples subjected to distillation.

### GC/MS and GC/FID analysis

Following the method reported in our previous studies [30, 35], GC–MS analysis was operated using an Agilent 7990B gas chromatograph coupled with a 5977A Agilent mass spectrometer. The gas chromatograph was equipped with an HP-5MS capillary column (5% phenylmethyl polysiloxane, 30 m length, 0.25 mm internal diameter, and 0.1 μm film thickness). The gradient temperature of the oven was programmed as follows: 5 min at 60^°^C, subsequently 3^°^C min^− 1^ to reach 180^°^C and for 1 min stop at 180^°^C. Furthermore, injector and transfer line temperatures were set at 230 and 240^°^C, respectively. Helium was used as carrier gas with a flow rate of 1 mL min^− 1^. The split ratio of the injector was 1:30, and the mass detector scanned through the range of 40–400 *m/z*. To identify EO compositions, a combinational trend was performed, including calculation of arithmetic retention indices using coherence of homologous series of *n*-alkanes (Supelco, Bellefonte, USA), matching the determined retention indices with those given in the reference publications [36], and the interpretation of mass data with the WILEY275 and NIST 05 libraries. As an additional procedure, the authenticity of some identified constituents was re-validated through certified reference standards (piperitenone oxide, α-pinene, β-pinene, sabinene, camphene, myrcene, and linalool from Supelco, Bellefonte, USA). Semi-quantitative analysis of EO compounds was carried out using an Agilent 7990B gas chromatograph (GC) equipped with a flame ionization detector (FID). The column used in the GC-FID device was VF-5MS which had the same stationary phase and dimensions as the HP-5MS. Likewise, the same thermal gradient program described above was used for the GC-FID analysis. Note that the EO samples were first diluted with *n*-hexane (1:100) before injection, and finally, an aliquot of 1 µL was used. For quantification, internal peak areas of each volatile constituent were integrated. The content of each compound was expressed as a relative percentage of the constituent present in the EO profile [37].

## Results and discussion

### Shoot and root dry weight accumulation

**Fig. 3:**
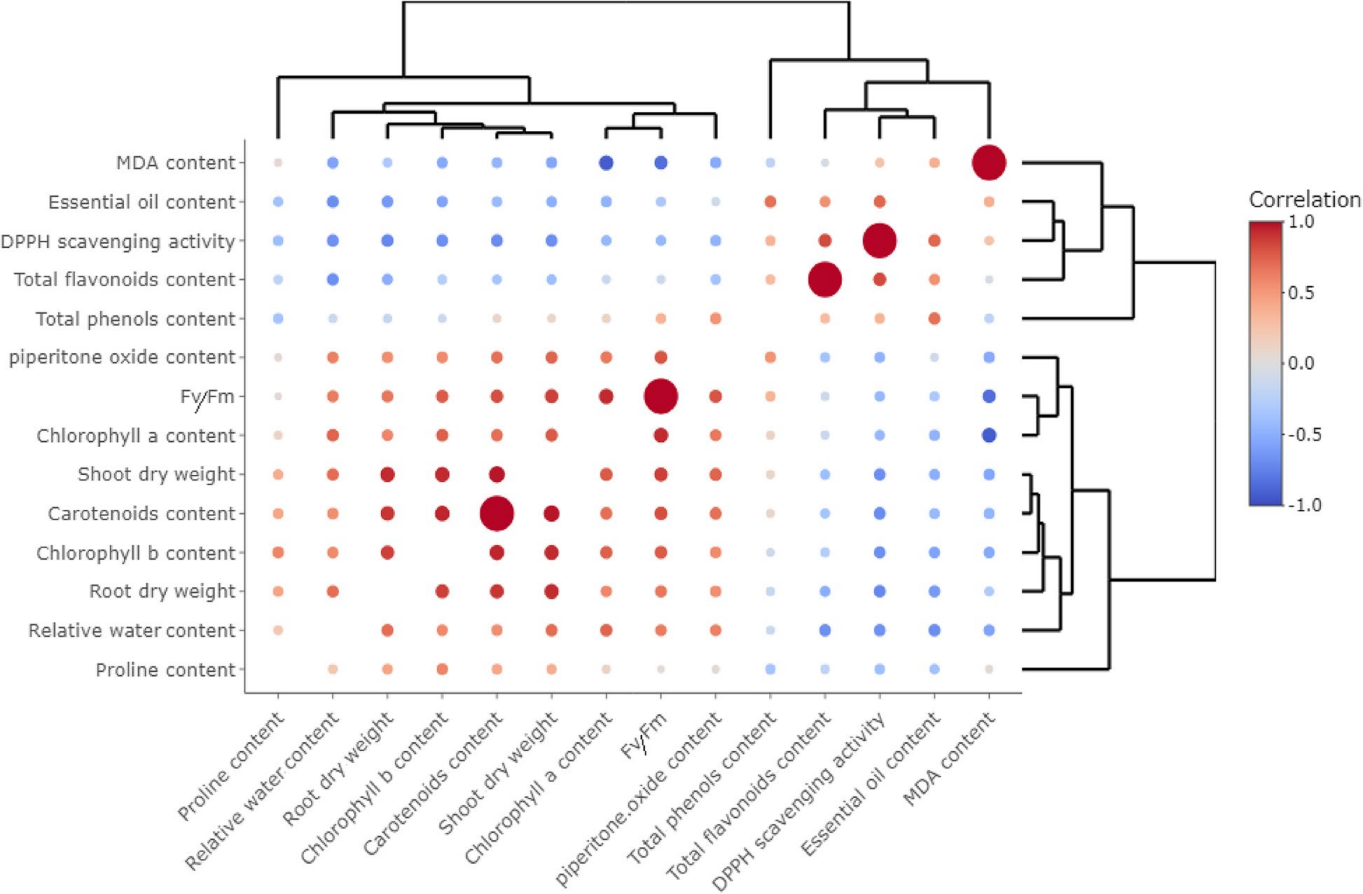
Interactive heatmap plot showing the relative correlations between measured characteristics. Note that the size of each point (circle) is based on –log_10_ (*p*-value). Hence, the greater circles have higher significance level

Table 1 shows the mean of shoots and roots dry weight obtained from the experiments following CCD configuration. A simple linear equation for actual input variables was generated for root and shoot dry weight targets by subjecting data to multiple regression analysis (Table 4). The regression analysis results are applicable to estimate the optimum value of independent variables to yield the maximum amount of biomass accumulation (Table 4). According to the analysis of variance (Table 2), both models developed for shoot and root dry weight were highly significant (*p* < 0.001). The effect of GABA and salt stress were significant at 1% level for shoot dry weight, whereas only salinity stress significantly (*p* < 0.01) affected root dry weight (Table 2). Salinity stress at 150 mM level harshly reduced shoot and dry root weight by 277 and 372%, respectively, compared with control (Table 1). According to Table 1; Fig. 1, the highest dry weight of shoots (30.33 ± 1.56 g) and roots (15.45 ± 2.60 g) was achieved under 2.4 mM GABA without NaCl application which does not have a significant difference with control treatment (i.e., 0 mM NaCl, 0 mM GABA). Optimization analysis predicted that under no-NaCl stress conditions, the application of 2.4 mM GABA would positively assist in achieving the highest yield of the shoot (32 g) and root dry weight (15 g) (Table 4). According to Fig. 3. there were interestingly positive and significant correlations between shoot/root dry weight and piperitenone oxide percentage, pigments content, relative water content, and maximum quantum yield of PSII. It can be inferred that a higher amount of biomass will be produced when the value of F_v_/F_m_ and photosynthetic pigments is enhanced. This correlation is in complete accordance with the results reported by Baghbani-Arani et al. [38], who declared that the health of photosynthetic systems, especially the light-harvesting units of PSII, is crucial for the economical production of higher biomass. Besides, some negative correlations were found between shoot/root dry weight and MDA content, total flavonoid content, and DPPH-scavenging activity. NaCl-induced osmotic damages and oxidative shocks perturb the normal metabolism of plant cells and restrict plant growth and biomass yield [14]. In this respect, Wu et al. [13] reported similar results by showing negative correlations between dry weight of tomato and MDA content, and H_2_O_2_ content as well. Previous literature showed that severe salinity can provoke a constant flux of reactive oxygen species (ROS), aggravating photosynthetic damages and diminishing the net photosynthetic rate [14]. This occurrence is then tightly linked to the reduced metabolism of endogenous GABA [19]. As proven by our and many other studies, plants pre-treated with exogenous GABA can rapidly recover from salinity injuries, and this result strongly acknowledges the irreplaceable importance of GABA in mitigating such harmful abiotic stresses [35, 39].

**Table 2:** Variance analysis of regression models fitting the indices related to drug yield and antioxidant properties, and coefficients for model precision

Traits	Piperitenone oxide		Essential oil content		Shoot dry weight		Root dry weight		Total phenols content		Total flavonoids content		DPPH-scavenging activity	
Model	Reduced cubic		Reduced quadratic		Linear		Linear		Reduced quartic		Reduced quartic		Reduced quartic	
	df	MS	df	MS	df	MS	df	MS	df	MS	df	MS	df	MS
Model	5	118.49^**^	4	0.13^**^	2	395.69^**^	2	166.36^**^	8	1568.25^**^	8	104.11^**^	8	1008.98^**^
X_1_ (NaCl)	1	252.66^**^	1	0.14^**^	1	728.59^**^	1	330.31^**^	1	0.17 ^ns^	1	342.23^**^	1	2388.61^**^
X_2_ (GABA)	1	94.95^**^	1	0.019^**^	1	62.78^**^	1	2.40^ns^	1	5.40 ^ns^	1	23.68^**^	1	715.96^**^
X_1_ × _2_	1	65.16^**^	1	0.067^**^	-	-	-	-	1	235.20^**^	1	11.27^*^	1	20.08 ^ns^
X_1_^2^	1	179.68^**^	1	0.31^**^	-	-	-	-	1	1733.16^**^	1	5.01 ^ns^	1	99.69 ^ns^
X_2_^2^	-	-	-	-	-	-	-	-	1	1716.52^**^	1	44.14^**^	1	505.23^**^
X_1_^2^ × _2_	1	90.52^**^	-	-	-	-	-	-	1	39.56 ^*^	1	3.85 ^ns^	1	780.55^**^
X_1_ × _2_^2^	-	-	-	-	-	-	-	-	1	13.92 ^ns^	1	222.27^**^	1	1182.30^**^
X_1_^3^	-	-	-	-	-	-	-	-	-	-	-	-	-	-
X_2_^3^	-	-	-	-	-	-	-	-	-	-	-	-	-	-
X_1_^2^ × _2_^2^	-	-	-	-	-	-	-	-	1	1914.27^**^	1	22.43^**^	1	299.99^*^
X_1_^3^ × _2_	-	-	-	-	-	-	-	-	-	-	-	-	-	-
X_1_ × _2_^3^	-	-	-	-	-	-	-	-	-	-	-	-	-	-
X_1_^4^	-	-	-	-	-	-	-	-	-	-	-	-	-	-
X_2_^4^	-	-	-	-	-	-	-	-	-	-	-	-	-	-
Residuals	24	8.92	25	0.002	27	6.87	27	2.56	-	-	-	-	-	-
Lack of fit	3	7.83^ns^	4	0.003^ns^	6	9.22^ns^	6	3.80 ^ns^	-	-	-	-	-	-
Pure Error	21	9.07	21	0.002	21	6.20	21	2.20	21	7.02	21	2.56	21	47.49
R^2^	0.73		0.89		0.81		0.82		0.99		0.93		0.89	
Adjusted R^2^	0.67		0.88		0.79		0.81		0.98		0.91		0.84	
Predicted R^2^	0.50		0.85		0.77		0.77		0.97		0.87		0.77	

*p < 0.05 and ** *p* < 0.01; ^ns^ means not significant

### Total polyphenols content, flavonoids content and DPPH radical scavenging activity

**Fig. 4:**
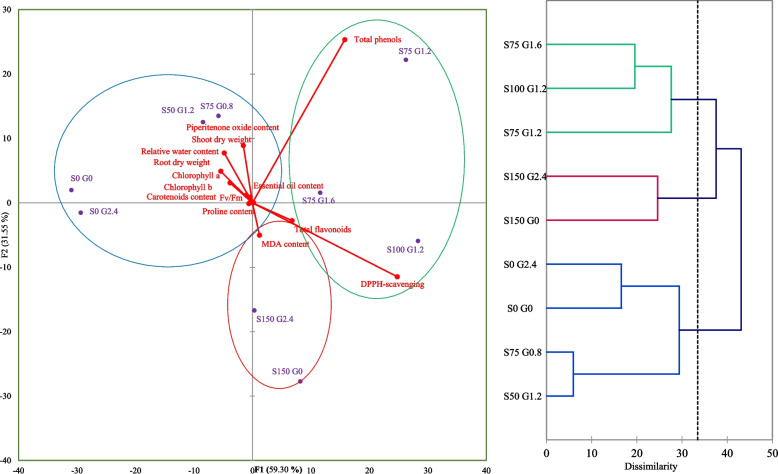
Principal component analysis (PCA) and cluster analysis (CA) reveal dissimilarities and group the effects of different treatments on measured traits. S represents salinity stress and G represents GABA application

The experimental values obtained for total polyphenols content (TPC), total flavonoids content (TFC), and DPPH radical scavenging activity were subjected to multiple non-linear regression analyses to fit a reduced fourth-order polynomial model (Tables 2 and 4). The developed models manifested high significance and satisfactory results with a low probability level (*p-value* < 0.01) and high R^2^ and F values, which implies that this mathematical model can predict dependent variables resultant from any combination of independent variables (Table 2). Except for total polyphenols content, both simple effects (GABA and salinity stress) were significant at 1% level. The interaction of simple terms (X_1_×X_2_) was significant for TFC (*p-value* < 0.05), and TPC (*p-value* < 0.01) variables. Unlike simple effects, the quadratic forms of simple effects (i.e., X_1_^2^and X_2_^2^) were significant at 1% probability level for the TPC variable. However, only the quadratic effect of X_2_^2^ was significant (*p-value* < 0.05) in developed models of TFC and DPPH- scavenging activity (Table 2). As presented in Table 2, the quadratic interactions and some other interactions were also significant in all three antioxidant-related traits (Table 2). Note that in these reduced models, the cubic and quadric terms were removed due to their low significance level to improve the reliability of the models. The 3D and contour response surface plot of total polyphenols content exhibited a dome-shaped curve with a high degree of convexity, with the maximum observed value of TPC around the central points of independent variables (Fig. 1, i & l). Likewise, the response surface plots related to TFC and DPPH variables indicated a well-defined, high grade of convexity closed to middle levels of GABA and at the endpoints of NaCl concentrations which could give the maximum response of dependent variables (Fig. 1, j, k, m, o). Compared to control, NaCl at 150 mM significantly ascended TFC and DPPH scavenging activity by about 36 and 115%, respectively (Table 1). In this regard, the study of Bistgani et al. [8] similarly showed the enhanced content of TPC, TFC, and phenolic acids under salinity in two *Thymus* species. By concurrent application of 100 mM NaCl and 1.2 mM GABA, TFC and DPPH- scavenging activity showed their highest levels being dramatically increased by about 74% (reaching 46.2 ± 0.57 mg QE g^− 1^ DW) and 154% (reaching 79.12 ± 1.47 mg QE g^− 1^ DW), respectively (Table 1). TPC was not significantly influenced by salinity stress compared with control (Table 1). However, by co-application of 75 mM NaCl and 1.2 mM GABA, TPC (191.27 ± 1.23 mg GAE g^− 1^ DW) was significantly enhanced by about 32% in comparison with control (144.51 ± 3.91 mg GAE g^− 1^ DW) and yielded the highest value among treatments (Table 1). It seems that within the range of 0.6–1.8 mM GABA and 75–100 mM NaCl, a higher value for TPC, TFC, and antioxidant activity can be expected (Figs. 1 and 4). Our previous study well matched the present study’s findings, which showed the increasing trends of flavonoids under drought-induced osmotic stress [9]. This study also showed that applying the amino acid citrulline at 2 mM and under severe water deficiency could considerably raise DPPH-scavenging activity. Citrulline acted as a compatible solute and free-radical scavenger by relieving dehydration stress [35]. Under water stress conditions, citrulline could decrease the plant’s metabolic energy expenditure to produce total polyphenols, as naturally synthesized antioxidants, and potentially osmolytes [10]. In most cases reported in the scientific papers, increased amounts of polyphenols and flavonoids under GABA application or other amino acids are mainly attributed to increased gene expression and activity of phenylalanine aminolyase (PAL) enzymes and also to decreased expression and activity of polyphenol oxidase (PPO) [17, 20, 25, 40]. Zarei et al. [16] showed that up-regulated expression of *CaM37* and other transcription factors (TFs) such as *WRKYs* and *MYBs* were the de facto reason for GABA involvement under salinity stress. The deployment of a reduced equation permitted a theoretical estimation of optimal conditions that were 75 and 1.2 mM, respectively, to reach the highest predicted value of TPC (191 mg GAE g^− 1^ DW) (Table 4). The optimal conditions for TFC (112 mM and 1.1 mM) and DPPH-radical scavenging activity (116 mM and 1.30 mM) are suggested to reach the highest predicted values (48 mg QE g^˗1^ DW and 80%). TFC was highly correlated with DPPH-radical scavenging activity (*r* = 0.83, *p-value* < 0.01) (Fig. 3). Further, TPC, TFC, and DPPH-radical scavenging activity were negatively correlated with root/shoot dry weight, pigments content, Fv/Fm, proline content and MDA concentration. Furthermore, positive correlations were found between these antioxidant-related characteristics and EO content (Fig. 3).

### Relative water, proline and malondialdehyde content

The values of RWC and MDA content obtained experimentally were analyzed by multiple regression analysis to fit a reduced cubic model (Table 3). Multiple regression analysis produced a reduced quartic model for total proline content (Table 3). All developed models were highly significant (*p-value* < 0.01) and provided satisfactory evidence of the goodness of fit tested through model summary statistics. All three stress-related models exhibited a high coefficient of determination (*R*^2^ > 0.90), indicating that the variation of dependent variables is properly explained by polynomial regression models (Table 3). When a model holds an excessive number of independent variables and polynomial terms, it becomes over-fitted, reflecting the irregularities and random noise [41]. Adjusted R-squared and predicted R-squared address both issues. Adjusted R-squared is used to compare the goodness of fit in regression models containing different numbers of independent variables [42]. The adjusted R-squared value rises when the new terms enhance the model’s statistical significance. In contrast, predicted R-squared helps determining if a regression model is overfitting [41]. The predicted R-squared value drastically drops when a model fits much more random noise than actual values [42]. A predicted R-squared distinctly smaller (greater than 0.2 difference) than R-squared is a forewarning sign of an overfitting model [42]. Fortunately, the developed models were efficiently adequate, as supported by a non-significant lack of fitness, high adjusted and predicted R^2^ values showing a short distance with R^2^. Adequate precision measures the signal-to-noise ratio. Its value should be greater than 4 in regression models. Fortunately, developed models of all measured characteristics had high values (> 5) of adequate precision (data are not shown). Except for the effect of GABA on MDA content, both simple effects (GABA and salinity stress) in these stress-related traits were significant at 1% probability level. Simple linear interactions (X_1_×X_2_) were also significant at 1% probability level for these indices (Table 3). The 3D surface plot and contour plot of these indices are reported in Fig. 2, showing change trends of these traits under salinity stress accompanied by application of *γ-*aminobutyric acid. The mean comparison results displayed that the relative water and proline content decreased under salinity stress (Table 1). Amassed proline and reduced content of cell’s relative water under environmental stresses is a well-characterized matter of stress physiology studies [9, 40, 43, 44]. Proline is a compatible solute and acts as a nitrogen repository in plant cells under environmental stresses [9]. Accumulated proline under salinity assists in recovering stress-induced oxidative damages through efficient ROS quenching and redox regulation [44]. MDA content was increased by gradually enhancing NaCl concentration (Table 1). MDA content under a single treatment of 150 mM NaCl reached the highest value (23.37 ± 1.69 mg g^− 1^ FW) compared with control (11.27 ± 0.77 mg g^− 1^ FW) (Table 1). Under normal conditions, foliar application of GABA at 2.4 mM did not significantly influence MDA content. The application of GABA achieved the lowest MDA content at its highest amount (2.4 mM) under the severest level of salinity stress (150 mM) (Table 1). The deployment of a reduced equation permitted a theoretical estimation of optimal conditions of 75 mM and 1.2 mM, respectively, to reach the highest predicted value of TPC (191 mg GAE g^− 1^ DW) (Table 4). Optimization analysis predicted similar values of independent variables to reach the lowest content of MDA (150 mM NaCl + 2.4 mM GABA) (Table 4). Moreover, the lowest proline content under 115 mM NaCl stress is achievable by applying 1.7 mM GABA (Table 4). Under moderate salinity stress, relative water content was predicted to be at its maximum point by applying 2.4 mM GABA (Table 4). Except for EO content and DPPH-scavenging activity, MDA content showed negative correlations with all experimental characteristics (Fig. 3). MDA content is considered as an important indicator of oxidative damage severity to biological membranes [9]. The results of the present study were in complete agreement with those of Sheteiwy et al. [40]. They stated that pre-application of GABA can inhibit the release of reactive oxygen species and decrease MDA levels, thus enhancing the cell membrane integrity under salinity-induced osmotic stress. In a parallel line with the findings of our study, Jin et al. [14] concluded that pre-spraying GABA under salinity-alkalinity stress decreased MDA content in muskmelon through modulation of antioxidant enzyme activity and redox balance. Our previous study well-concurred with the outcomes of this study by showing that application of the amino acid citrulline as an antioxidant defense inducer can reduce H_2_O_2_ content and other possible oxidative reactions, thus preventing the formation of lipid peroxidation products such as MDA [9]. Another work supported that the accumulation of amino acids such as GABA in leaves may induce the formation of strong hydrogen-bonded water around proteins and thus protect the innate arrangement of proteinogenic polymers of membranes, to diminish MDA amount [13].

**Table 3:** Variance analysis of regression models fitting physiological and biochemical indices and coefficients for model precision

Traits	Relative water content (%)		Proline content (mg g^− 1^ FW)		MDA content (mg g^− 1^ FW)		Chlorophyll a content (mg g^− 1^ FW)		Chlorophyll b content (mg g^− 1^ FW)		Carotenoids content (mg g^− 1^ FW)		Fv/Fm ratio	
Model	Reduced cubic		Reduced quartic		Reduced cubic		Quadratic		Reduced cubic		Linear		Quadratic	
	df	MS	df	MS	df	MS	df	MS	df	MS	df	MS	df	MS
Model	6	131.89^**^	8	2.52^**^	5	126.40^**^	5	2.85^**^	7	0.34^**^	2	0.041^**^	5	0.011^**^
X_1_ (NaCl)	1	56.51^**^	1	0.95^**^	1	51.81^**^	1	4.62^**^	1	0.0049 ^ns^	1	0.072^**^	1	0.025^**^
X_2_ (GABA)	1	8.42^**^	1	0.41^**^	1	0.24 ^ns^	1	3.46^**^	1	0.032 ^ns^	1	0.0096^**^	1	0.011^**^
X_1_ × _2_	1	342.72^**^	1	0.70^**^	1	310.59^**^	1	5.54^**^	1	0.0088 ^ns^	-	-	1	0.0085^**^
X_1_^2^	1	13.93^**^	1	6.76^**^	1	30.19^**^	1	0.32 ^*^	1	0.11^**^	-	-	1	0.00005^ns^
X_2_^2^	1	19.12^**^	1	0.084 ^ns^	-	-	1	0.23 ^ns^	1	0.13^**^	-	-	1	0.00004^ns^
X_1_^2^ × _2_	-	-	1	0.53^**^	1	9.43^**^	-	-	1	0.11^**^	-	-	-	-
X_1_ × _2_^2^	1	9.20^**^	1	1.82^**^	-	-	-	-	1	0.13^**^	-	-	-	-
X_1_^3^	-	-	-	-	-	-	-	-	-	-	-	-	-	-
X_2_^3^	-	-	-	-	-	-	-	-	-	-	-	-	-	-
X_1_^2^ × _2_^2^	-	-	1	2.03^**^	-	-	-	-	-	-	-	-	-	-
X_1_^3^ × _2_	-	-	-	-	-	-	-	-	-	-	-	-	-	-
X_1_ × _2_^3^	-	-	-	-	-	-	-	-	-	-	-	-	-	-
X_1_^4^	-	-	-	-	-	-	-	-	-	-	-	-	-	-
X_2_^4^	-	-	-	-	-	-	-	-	-	-	-	-	-	-
Residuals	23	1.00	-	-	24	1.05	24	0.068	22	0.0051	27	0.0005	24	0.00046
Lack of fit	2	1.54 ^ns^	-	-	3	0.99 ^ns^	3	0.15 ^ns^	1	0.0013 ^ns^	6	0.001^ns^	3	0.00034 ^ns^
Pure Error	21	0.95	21	0.029	21	1.06	21	0.056	21	0.0053	21	0.0004	21	0.00048
R^2^	0.97		0.97		0.96		0.89		0.95		0.83		0.82	
Adjusted R^2^	0.96		0.96		0.95		0.87		0.93		0.82		0.79	
Predicted R^2^	0.95		0.93		0.93		0.83		0.92		0.78		0.73	

*p < 0.05 and ** *p* < 0.01; ^ns^ means not significant

**Table 4:** Model equation in terms of actual factors for measured indices and optimized levels of each factor needed to achieve maximum or minimum Response

Trait	Model	Model equation in terms of actual factors	Optimized levels of factors (Salinity stress and GABA)	Targeted response	Optimized response
Piperitenone oxide content	Reduced cubic	*R* = 74.73 -0.36 × _1_-1.9 × _2_ -0.24 × _1_ × _2_-0.003 × _1_^2^ + 0.0017 × _1_^2^ × _2_	95 mM and 0.2 mM	Maximum	81%
Essential oil content	Reduced quadratic	*R* = 0.17 + 0.008 × _1_ + 0.030 × _2_-0.0008 × _1_ × _2_-0.0003 × _2_	100 mM and 0.1 mM	Maximum	0.6%
Shoot dry weight	Linear	*R* = 27.26-0.10 × _1_ + 1.85 × _2_	0 mM and 2.4 mM	Maximum	32 g
Root dry weight	Linear	*R* = 13.81-0.06 × _1_ + 0.36 × _2_	0 mM and 2.4 mM	Maximum	15 g
Total phenols content	Reduced quartic	*R* = 124.50-4.01 × _1_-258.02 × _2_ + 16.53 × _1_ × _2_ + 0.02 × _1_^2^ + 110.80 × _2_^2^-0.11 × _1_^2^ × _2_ -6.98 × _1_ × _2_^2^ + 0.046 × _1_^2^ × _2_^2^	75 mM and 1.2 mM	Maximum	191 mg GAE g^− 1^ DW
Total flavonoids content	Reduced quartic	*R* = 26.51-0.78 × _1_-38.47 × _2_ + 2.28 × _1_ × _2_+ 0.005 × _1_^2^ + 17.58 × _2_^2^-0.012 × _1_^2^ × _2_-0.93 × _1_ × _2_^2^ + 0.005 × _1_^2^ × _2_^2^	112 mM and 1.1 mM	Maximum	48 mg QE g-^1^ DW
DPPH-scavenging Activity	Reduced quartic	*R* = 31.06-3.32 × _1_ -128.77 × _2_ + 8.40 × _1_ × _2_ + 0.023 × _1_^2^ + 53.27 × _2_^2^ -0.049 × _1_^2^ × _2_ -3.18 × _1_ × _2_^2^ + 0.018 × _1_^2^ × _2_^2^	116 mM and 1.30 mM	Maximum	80%
Relative water content (%)	Reduced cubic	*R* = 88.64 + 0.11 × _1_ -17.75 × _2_ -0.025 × _1_ × _2_-0.0017 × _1_^2^ + 5.261 × _2_^2^ + 0.035 × _1_ × _1_^2^	75 mM and 2.4 mM	Maximum	86%
Proline content (mg g^− 1^ FW)	Reduced quartic	*R* = 6.65 + 0.051 × _1_ -25.63 × _2_ + 0.56 × _1_ × _2_ -0.0036 × _1_^2^ + 10.81 × _2_^2^ -0.0035 × _1_^2^ × _2_ -0.24 × _1_ × _2_^2^ + 0.0015 × _1_^2^ × _2_^2^	115 mM and 1.7 mM	Minimum	3.35 mg g^− 1^ FW
MDA content (mg g^− 1^ FW)	Reduced cubic	*R* = 11.22-0.06 × _1_ + 0.51 × _2_ + 0.029 × _1_ × _2_ + 0.001 × _1_^2^-0.0005 × _1_^2^ × _2_	150 mM and 2.4 mM	Minimum	6.35 mg g^− 1^ FW
Chlorophyll a content (mg g^− 1^ FW)	Quadratic	*R* = 4.08-0.022 × _1_-2.19 × _2_ + 0.0075 × _1_ × _2_ + 0.00026 × _1_^2^-0.861 × _2_^2^	70 mM and 2.4 mM	Maximum	5.3 mg g^− 1^ FW
Chlorophyll b content (mg g^− 1^ FW)	Reduced cubic	*R* = 1.4 + 0.029 × _1_-2.20 × _2_ + 0.0009 × _1_ × _2_-0.0002 × _1_^2^ + 0.97 × _2_^2^ + 0.00006 × _1_^2^ × _2_ -0.004 × _1_ × _2_^2^	70 mM and 0.25 mM	Maximum	1.93 mg g^− 1^ FW
Carotenoids content (mg g^− 1^ FW)	Linear	*R* = 0.296-0.001 × _1_ + 0.022 × _2_	0 mM and 2.4 mM	Maximum	0.35 mg g^− 1^ FW
Fv/Fm ratio	Quadratic	*R* = 0.76-0.0004 × _1_ + 0.030 × _2_ + 0.00029 × _1_ × _2_-0.50 × _1_^2^-0.011 × _2_^2^	0 mM and 1.3 mM	Maximum	0.79

### Essential oil contents and constituents

The EO content of 30 experimental samples varied from 0.15 to 0.60% (v/w). GC–MS analysis characterized 15 EO compositions allowing to identify a total percentage of 78.81–91.51%. The major compounds were piperitenone oxide (ranging from 58.80 to 79.71%), viridiflorol (0.21–15.81%), germacrene D (0.25–9.23%), *β*-farnesene (0.68–4.59–4.74%), *β*-elemene (0.92–4.84%) and limonene (0.70–4.74%). Multiple non-linear regression analyses developed a reduced third order and a reduced second order model for piperitenone oxide and EO content, respectively (Table 2). Both models were statistically significant (*p-value* < 0.01) and had relatively high R-squared (> 0.7) and Fisher values. Additionally, a non-significant lack of fitness provided further approval for model trustworthiness, which shows high reliability and accuracy (Table 2). Multiple regression analysis revealed that linear terms and their interactions affect these traits. The second-order term of NaCl stress (X_1_^2^) affected also the EO and piperitenone content (*p-value* < 0.01). The quadratic × linear interaction of NaCl and GABA (X_1_^2^ × _2_) in the cubic model significantly influenced the piperitenone oxide variable at 1% probability. The three-dimensional surface and contour plot of EO content demonstrate that the highest value is attainable under moderate to severe NaCl stress (60–150 mM), whether combined with GABA (up to 1.2 mM) or not (Fig. 1c, g). However, 3D and contour plots showed that piperitenone oxide content was substantially reduced under severe salinity stresses (under concentrations more than 120 mM NaCl) (Fig. 1d, h). Furthermore, the results showed that applying GABA (up to 0.8 mM) under moderate salinity stresses (50–100 mM) can give a high piperitenone oxide content (Fig. 1d, h). The amount of EO (0.18 ± 0.03%) under 150 mM NaCl stress was three times greater than that of control (0.53 ± 0.06%) (Table 1). However, severe salinity stress (150 mM NaCl) yielded the lowest piperitenone content, and a 20% reduction (61.17 ± 3.7%) when compared with control (Table 1). Foliar application of GABA elicited the production of EO and its main component (i.e., piperitenone oxide). Under 75 mM NaCl stress, GABA application at 0.8 mM gave the highest amount of both EO and piperitenone oxide (Table 1; Fig. 4). Due to its unique properties, plants rich in piperitenone oxide are constantly used in cosmetics, pharmaceuticals, beverages, tobacco, and insect-repellent products. Hence, over-production of piperitenone oxide-rich EOs using metabolic elicitors will be the best approach to acquiring much more economic profits. Optimization analysis suggested a frugal consumption of GABA under 100 mM NaCl stress to yield the maximum content of EO (0.6% at 0.1 mM GABA) and piperitenone oxide (81% at 0.2 mM GABA) (Table 4). Interestingly, correlation analysis unraveled a positive correlation between EO content and stress-related indices. In contrast, piperitenone oxide content was negatively correlated with stress indices (Fig. 3). When abiotic stress perturbs the CO_2_ sequestration, the diverted biosynthesis pathways re-allocate excessive NADPH reductants to produce secondary metabolites. The switched metabolic path accompanied by increased amounts of stress-related products (e.g., EOs, polyphenols) may contribute to an enhanced tolerance mechanism of the plant [6]. In support of our findings, the investigation of Liao et al. [45] on tea showed that GABA elicits gene expressions and the biosynthetic pathway of flavonoids. In another confirmation, the eliciting effects of salicylic acid and chitosan as metabolic agents on the production of secoiridoid and xanthone glycosides in *Swertia paniculata* Wall were proved [27]. Our recent publication on hyssop (*Hyssopus officinalis* L.) also demonstrated that foliar application of citrulline could contribute to an increased isopinocamphone content under water deficiency [9]. These results demonstrated that GABA could strongly protect tomato seedlings against NaCl stress [13]. Plant resistance against salinity depends on a complicated regulatory genes network, transcription factors, enzymes, and plant physiology [7]. This study’s fluctuation of secondary metabolites elucidated that this change is due to the plant’s need for defense-inducing molecules to help them surviving in unfavorable conditions.

### Pigments content and chlorophyll fluorescence

Chlorophyll is a prerequisite for mobilizing photosynthetic machines [13]. Decreased production of photosynthetic pigments and their reduced capacity is an essential determining factor for plant growth reduction when subjected to environmental stresses [10]. Higher chlorophyll content enables plants to capture more light energy and conversion efficiency, thereby improving photosynthetic capacity [13]. Data obtained from measurement of chlorophyll *a*, *b*, carotenoids content, and maximum quantum yield of PSII (Fv/Fm ratio) fitted to quadratic, reduced cubic, linear and quadratic models, respectively (Table 3). The developed models were significant at 1% probability level and provided satisfying results by showing relatively high R^2^ (Table 3). Except for chlorophyll *b*, linear terms significantly affected the traits mentioned above (Table 3). The interaction of simple terms (X_1_×X_2_) was significant for chlorophyll *a* and F_v_/F_m_ ratio (*p-value* < 0.01) (Table 3). The second-order effect of NaCl significantly affected chlorophyll *a* at 5% level (Table 3). Moreover, the quadratic terms (X_1_^2^ and X_2_^2^) and quadratic × linear interactions of NaCl and GABA (X_1_^2^ × _2_ and X_1_ × _2_^2^) in the developed model for chlorophyll *b* were significant at 1% probability level (Table 3). As presented in Table 4, optimization analysis predicted different solutions to reach the maximum quantity of pigments content and F_v_/F_m_ ratio. The severest salinity stress (150 mM) reduced the amount of chlorophyll *a*, chlorophyll *b*, carotenoids, and maximum quantum yield of PSII by about 64, 50, 57, and 20%, respectively. By disregarding control treatment, the highest values of these traits were acquired by applying 2.4 mM GABA under non-stressed conditions. GABA efficiently could prohibit chlorophyll degradation and reduction of maximum quantum yield of PSII under different concentrations of salinity stress. The investigation of Sheteiwy et al. [40] on rice assented to the finding of the present study. The study deciphered that GABA priming can inhibit the reduction of photosynthetic parameters and pigments content, whether used singly or combined with salinity and osmotic stress. The study of Wu et al. [13] on tomatoes was also well-consistent with the results of this study by showing that exogenous GABA could delay the reduction of chlorophyll levels under NaCl stress. On the contrary, another research showed that under non-stress condition, pre-application of GABA limits the transformation from chlorophyll *a* to chlorophyll *b*, which results in higher chlorophyll accumulation and lower chlorophyll *b* concentration [14]. The authors also indicated that chlorophyll contents sharply enhanced under salinity-alkalinity stress, while GABA pretreatment reversed these trends and reduced related gene expression patterns [14]. They inferred in this sense that over-accumulation of chlorophyll and its precursors under salinity stress might trigger photo-oxidation injury, which ultimately can cause membrane damage [14]. Under salinity stress, photosystem proteins are denatured, the electron transport chain disturbed, and thus the redox imbalance can be found in abundance [46]. In this condition, instead of photochemical quenching, non-photochemical quenching (xanthophyll cycle) will be responsible for lowering photo-oxidative damages [46]. Recent studies confirm that environmental stresses can inevitably descend the level of photosynthetic efficiency (F_v_/F_m_ ratio) [7, 10]. As illustrated in Fig. 3, pigments content and F_v_/F_m_ ratio were positively correlated with shoot/root dry weight, RWC, proline content, and piperitenone oxide percent. These traits were also negatively correlated with the contents of EO, MDA, and flavonoids (Fig. 3).

## Conclusions

Based on the results, extremely severe NaCl stresses (i.e., more than 100 mM) were out of *M. suaveolens* salinity tolerance threshold. However, under moderate salinity (i.e., 50–100 mM), majority of stress related indices (total polyphenols, total flavonoids, DPPH-radical scavenging, relative water content, proline content, pigments content and maximum quantum yield of PSII) reached a desired value to alleviate salt stress by concurrent application of midpoint levels of GABA (i.e., 0.8–1.6 mM). Optimization analysis predicted that under 100 mM or lower levels of NaCl stress, use of a dilute γ-amino-butyric acid solution (i.e., 0.1–0.2 mM) ill positively affects shoot dry weight, EO content and piperitenone oxide amount to obtain the highest values. Therefore, the safe application of GABA not only seems economic, but can also provide the possibility for a high-throughput production of pineapple mint as an essential oil-bearing medicinal plant in saline soils.

## Supplementary Information

**Additional file 1. Table S1.** Physio-chemical properties and texture of field soil used in the experiment. **Fig. S1.** GC-MS chromatogram of pineapple mint essential oil. **Fig. S2.** Picture 1 shows the pineapple mint treated with NaCl without GABA application. Picture 2 shows the pineapple mint treated with NaCl and GABA. Optimum concentration of GABA reduces the effect of NaCl.

## Data Availability

All the data generated or analyzed during the current study were included in the manuscript. The raw data is available from the corresponding author on reasonable request.
