# Supplementary material for: Modeling and optimizing concentration of exogenous application of γ-aminobutyric acid on NaCl-stressed pineapple mint (Mentha suaveolens) using response surface methodology: an investigation into secondary metabolites and physiological parameters
Source: BMC Plant Biol. 2023 Jun 10;23:309. doi: 10.1186/s12870-023-04312-w (PMC10257319; doi:10.1186/s12870-023-04312-w)
Supplement: Supplementary file 1 — Additional file 1. Table S1. Physio-chemical properties and texture of field soil used in the experiment. Fig. S1. GC-MS chromatogram of pineapple mint essential oil. Fig. S2. Picture 1 shows the pineapple mint treated with NaCl without GABA application. Picture 2 shows the pineapple mint treated with NaCl and GABA. Optimum concentration of GABA reduces the effect of NaCl. [file 12870_2023_4312_MOESM1_ESM.docx]

| Properties | Value |
| --- | --- |
| Sand (%) | 74.03 |
| Silt (%) | 20.76 |
| Clay (%) | 5.19 |
| Organic carbon (%) | 18.7 |
| CaCo_3_ (%) | 62.25 |
| Fe (mg kg^-1^) | 4.15 |
| Zn (mg kg^-1^) | 0.45 |
| Cu (mg kg^-1^) | 0.15 |
| Mn (mg kg^-1^) | 1.68 |
| P (mg kg^-1^) | 42.71 |
| K (mg kg^-1^) | 50.70 |
| EC (ds m^-2^) | 0.48 |
| pH | 7.50 |

**Table S1**. Physio-chemical properties and texture of field soil used in the experiment.


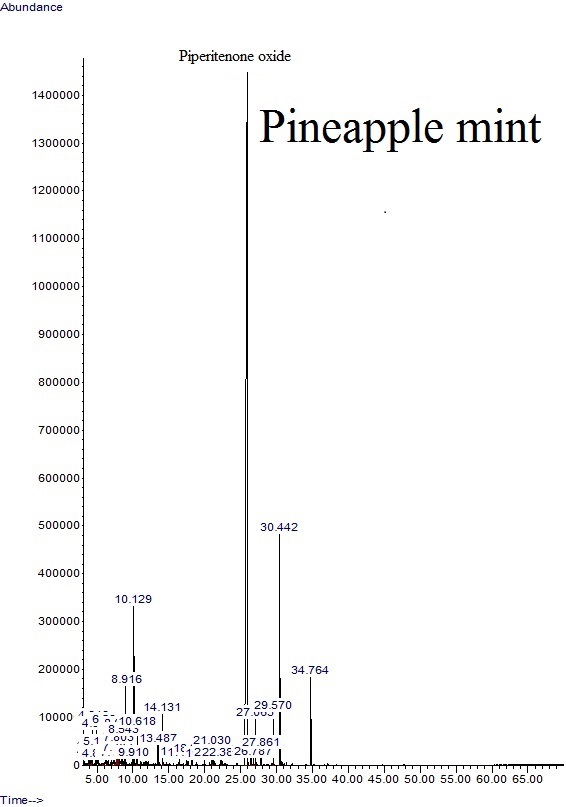


Fig. S1. GC-MS chromatogram of pineapple mint essential oil


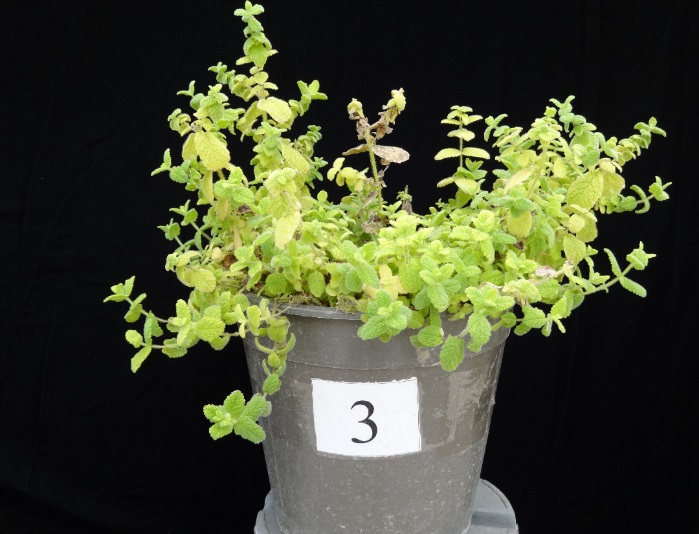

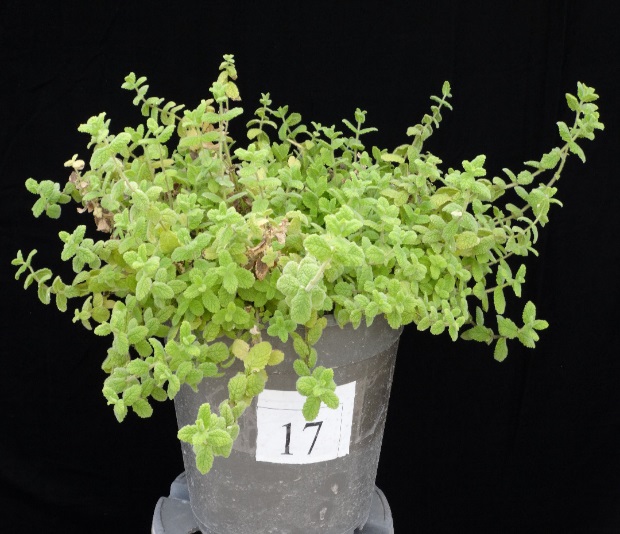


2

1

Fig. S2. Picture 1 shows the pineapple mint treated with NaCl without GABA application. Picture 2 shows the pineapple mint treated with NaCl and GABA. Optimum concentration of GABA reduces the effect of NaCl.
